# Pilot-Scale Optimization of Supercritical CO_2_ Extraction of Dry Paprika *Capsicum annuum*: Influence of Operational Conditions and Storage on Extract Composition

**DOI:** 10.3390/molecules27072090

**Published:** 2022-03-24

**Authors:** Dorota Kostrzewa, Agnieszka Dobrzyńska-Inger, Barbara Mazurek, Marcin Kostrzewa

**Affiliations:** 1 Łukasiewicz Research Network—New Chemical Syntheses Institute, Al. Tysiąclecia Państwa Polskiego 13A, 24-110 Pulawy, Poland; agnieszka.dobrzynska-inger@ins.lukasiewicz.gov.pl (A.D.-I.); barbara.mazurek@ins.lukasiewicz.gov.pl (B.M.); 2 Faculty of Chemical Engineering and Commodity Science, Kazimierz Pulaski University of Technology and Humanities in Radom, Chrobrego 27, 26-600 Radom, Poland; m.kostrzewa@uthrad.pl

**Keywords:** dry red paprika, supercritical carbon dioxide extraction, carotenoids, fatty acids, response surface methodology

## Abstract

Supercritical carbon dioxide extraction was used to extract carotenoids from dry paprika *Capsicum annuum*. Studies regarding the effect of process parameters, including pressure (25–45 MPa), temperature (40–60 °C), and time (10–110 min), were carried out using response surface methodology. It was found that under optimal conditions (pressure of 45 MPa, temperature of 50 °C, and time of 74 min), the extract yield was 10.05%, and the total content of carotenoids in the extract was 4.21%, in good agreement with the predicted values (10.24% and 4.24%, respectively). Composition analysis showed that paprika extract mainly consisted of linoleic acid. There was no significant difference between the fatty acid content of the extracts obtained by SC-CO_2_ extraction and n-hexane Soxhlet extraction. For functional purposes, the effect of storage conditions and time on the quality of paprika extract was also specified.

## 1. Introduction

Carotenoids are applied as natural dyes, and they also have many other bioactivities such as antioxidant [1,2], antitumor [3,4], and antiadipogenic [5]. Therefore, there is a growing interest in these compounds in various industries. The global carotenoid market is estimated to grow from USD 1.5 billion in 2019 to USD 2.0 billion in 2026 [6]. Currently, there is a visible tendency to obtain these compounds from natural sources such as fruit and vegetables [1,7]. This has been supported by consumer preferences and health benefits [1,8,9]. The active forms of natural carotenoids significantly exceed the effectiveness of compounds of a synthetic origin. Moreover, they do not show toxic or carcinogenic effects [8].

**Figure 1:**
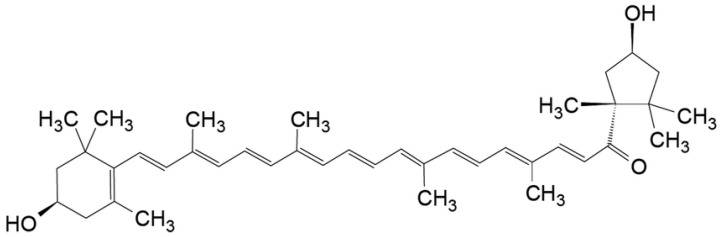
Structure of capsanthin, major carotenoids in red paprika *Capsicum annuum* L.

An excellent source of these phytochemicals is red paprika *Capsicum annuum* L. [1,9,10,11,12,13,14]. The major carotenoid found in red paprika is capsanthin (Figure 1), and it also contains capsorubin, β-carotene, zeaxanthin, violaxanthin, β-cryptoxanthin, and lutein [14]. According to literature reports, carotenoids present in paprika are precursors of vitamin A [15]. They are effective as free radical scavengers, have a positive effect on the cholesterol level in plasma [12], and showing significant activity of neutralization of singlet oxygen and inhibiting effect on lipid peroxidation [16]. Carotenoids reduce the risk of cardiovascular and chronic diseases by providing eye protection [1,11,17,18,19].

Additionally, paprika contains vitamins E, C, and B, pro-vitamin A, phenolic compounds, fats, and micro- and macroelements. The content of oil containing essential unsaturated fatty acids in ground paprika is in the range of 10–14% [20]. Recent studies have shown that oleic acid present in paprika can have a positive effect on cholesterol levels, reducing the risk of cardiovascular disease, and the high content of linoleic acid makes it a product of nutritional value.

In general, to obtain valuable paprika components, organic solvent extraction [21,22] and nonconventional extraction methods such as microwave-assisted extraction (MAE) [23] and ultrasound-assisted extraction (UAE) [21] are applied. However, for these techniques, long extraction time, lack of selectivity and remains of toxic solvents in the final product, energy-intensive separation of solvent, and degradation of compounds sensitive to temperature are observed [24]. These drawbacks can be avoided using the green technique of supercritical carbon dioxide (SC-CO_2_) extraction. Carbon dioxide is a solvent with low critical parameters, inexpensive, nontoxic, nonexplosive, and easily removable from the final product.

Although researchers have already presented SC-CO_2_ extraction, most reports focus on extracting capsaicinoids from hot paprika [25,26,27,28,29,30]. Frequently, the presented studies refer to a very small scale of the process (extractor’s volume of 50 cm^3^) [25,26,28,31,32,33]. Many authors report only the concentration of carotenoids in paprika extracts without concluding on the efficiency of carotenoid recovery, which is very important for process economics. There is a lack of information on optimal parameters for obtaining carotenoids using SC-CO_2_ from dry paprika. This is important because previous studies have shown that the water content in raw materials has a negative impact on the amount of carotenoids obtained [34]. There are also a lack of data determining the stability of carotenoids in extract during storage.

Therefore, the purpose of this study was to investigate the SC-CO_2_ extraction of carotenoids from dried paprika under different pressure and temperature conditions. The process was carried out in a pilot-scale plant with the closing of carbon dioxide circulation. This will make the results easier to transfer to an industrial process. For the studies, advanced methods of design of experiments (DOE) and the response surface method (RSM) were used. This allowed gaining more in-depth knowledge of the process and specifying optimal conditions for obtaining extracts rich in bioactive compounds. In order to increase the utility value of the extracts, they were analyzed for their fatty acid content. The results obtained with SC-CO_2_ were compared to the results of paprika extraction using the organic solvents and SFE results of paprika with higher moisture content. Further, the influence of storage conditions and time on the stability of carotenoids in pepper extracts was determined.

## 2. Materials and Methods

### 2.1. Materials

Ground red paprika *Capsicum annuum* L. was purchased from P.H. Royal Sp. z o.o. (Poland). The color value was 165 ASTA (American Spice Trade Association color value). The sieve analysis performed using the LPzE-2e laboratory sieve shaker (Multiserw—Morek Company, Marcyporęba, Poland) showed that the average particle size of the raw material was 0.27 mm. Before extraction, paprika was air-dried for 2 h at 50 °C. After that, the raw material moisture content determined using the Radwag MAC 50/1 analyzer amounted to 7.5 ± 0.2%. This is the optimal humidity determined during our previous studies [34].

Carbon dioxide (99.9% purity) was purchased from Grupa Azoty Zakłady Azotowe “Puławy” S.A. (Puławy, Poland). All organic solvents and reagents were of analytical or GC purity and were purchased from Sigma-Aldrich Co. (St. Louis, MO, USA) and Avantor Performance Materials Poland S.A. (Gliwice, Poland). Analytical standards of methyl esters of fatty acids were provided by Sigma-Aldrich Co. (St. Louis, MO, USA).

### 2.2. Solvent Extraction (SOX)

A 30 g amount of ground paprika and 300 mL of n-hexane were placed in a Soxhlet apparatus and kept in a gentle boil for 4 h (drop flow of 140 drops/min). After extraction, n-hexane was removed using a rotary evaporator (Rotavapor R-210, Labortechnik AG Büchi, Flawil, Switzerland), and then the weight of the extract was measured gravimetrically. Extraction of paprika was repeated three times. The obtained extracts were stored at 5 °C until analysis. The extraction yield was expressed as a percentage and defined as the weight of extract over the weight of feedstock.

### 2.3. Supercritical Carbon Dioxide (SC-CO_2_) Extraction

All experiments were conducted in a SITEC pilot plant (SITEC-Sieber Engineering AG, Maur (Zurich), Switzerland). For all runs, the extractor was loaded with approximately 200 g of ground paprika. Then, the vessel was filled with carbon dioxide, and the pressure was increased to the pressure in the condenser. In the next stage, the pressure in the extraction system was increased using a high-pressure pump. After achieving the required extraction parameters, the flow of carbon dioxide through the extractor was started. The extract samples were taken from the separator, placed into the collecting vessel at constant time intervals (5 min), and weighed each time to attain the kinetics of extraction and to specify the final yield of extraction. After extraction, the system was switched back to the bypass to wash out extract deposits left in the plant (20 min). Then, the pressure in the extractor was lowered to the atmospheric value, the extractor was opened, and the post-extraction residue was removed. The extraction yield was expressed in g of extract per g of sample (×100%). The extract’s apparent solubility was calculated from the linear part of the extraction curves and expressed in g of extract per kg of solvent.

### 2.4. Design of Experiments

The response surface method (RSM) was applied to study the extraction of dry paprika with SC-CO_2_ and to optimize the process. The effect of pressure (X_1_: 25–45 MPa), temperature (X_2_: 40–60 °C), and extraction time (X_3_: 10–110 min) on the yield and quality of the extract was analyzed using the three-level Box–Behnken design. The design matrix indicating the actual-coded independent variables is presented in Table 1. The analyzed response variables were: extraction yield (Y_e_), color value of extract (B_e_), total content of carotenoids in extract (C_k_), concentration of red carotenoid fraction in extract (C_r_), concentration of yellow carotenoid fraction in extract (C_y_), and color value of post-extraction residue (B_p_). The effect of independent variables on the response variables was approximated with the second-order polynomial regression model expressed by Equation (7).

(1) $$Y=b_{0}+\sum_{i=1}^{n}b_{i}x_{i}+\sum_{i=1}^{n}\left(b_{ii}x_{i}{}^{2}\right)+\sum_{i=1}^{n}\sum_{j=1;i<j}^{n}b_{ij}x_{i}x_{j}$$

where *Y* is the response variable; *x_i_* and *x_j_* are independent variables; and *b*_0_, *b_i_*, *b_ii_*, and *b_ij_* are coefficients in the equation referring to equation constant, major effects of parameters, and their interactions.

For multiple regression analysis, analysis of variance (ANOVA) was used, and for optimization of the process, Design Expert 9.0.6.2 (Stat-Ease Inc., Minneapolis, MN, USA) was used. The analysis includes the F test (overall model significance), its associated probability P(F), and lack of fit (LOF). The coefficient of determination R^2^, adjusted coefficient of determination R^2^, and predicted coefficient R^2^ were applied to evaluate the accuracy of fit between experimental data and the model. The *p*-value was used to evaluate the significance of the model coefficients. The statistical significance test was based on the total error criteria with a confidence level of 95%.

### 2.5. Characteristics of Raw Material and Paprika Extract

The total content of carotenoids in the raw material and extract, color value of extract, and post-extraction residue were determined according to the procedure described in Compendium of Food Additive Specifications [35] and the Official Analytical Methods of the American Spice Trade Association [36]. The concentration of red and yellow fractions of carotenoids was determined according to the procedure described by Fernandez-Ronco et al. [37]. These methods are based on absorbance measurements of acetone solute solution with an appropriate wavelength. The absorbance of the solution was measured using the JASCO V-650 UV-Vis spectrophotometer (JASCO International Co., Ltd., Tokyo, Japan). A detailed description of the methodology was presented in a previous paper [38]. Each measurement was repeated three times.

### 2.6. Determination of Fatty Acids

The content of fatty acids in the extract was determined using gas chromatography following the methodology presented in a previous paper [39]. This method involves the indirect determination of fatty acids in the form of methyl esters obtained by esterification with trimethylsulfonium hydroxide solution. For the analysis, the Agilent Technologies 6890 N chromatograph coupled with the MSD 5975 mass spectrometer was used. The applied carrier gas was helium with a flow from 1.0 to 1.5 mL/min. Measurements were carried out for the following parameters: HP-88 column (60 m, 0.25 mm i.d., 0.20 μm film thickness), inject volume of 1 μL, scan range of 50–500 amu, quadrupole temperature of 150 °C, ion source temperature of 230 °C. The spectrometer was operated in ‘full scan’ mode with ionization energy of 70 eV.

The qualitative analysis was based on the mass spectra available from the NIST Research Library and on the comparison of retention time peaks with appropriate standards. The content of individual FAs was calculated by the external standard method and expressed as weight percentages. Each measurement was repeated three times.

## 3. Results and Discussion

### 3.1. Extraction Kinetics

**Figure 2:**
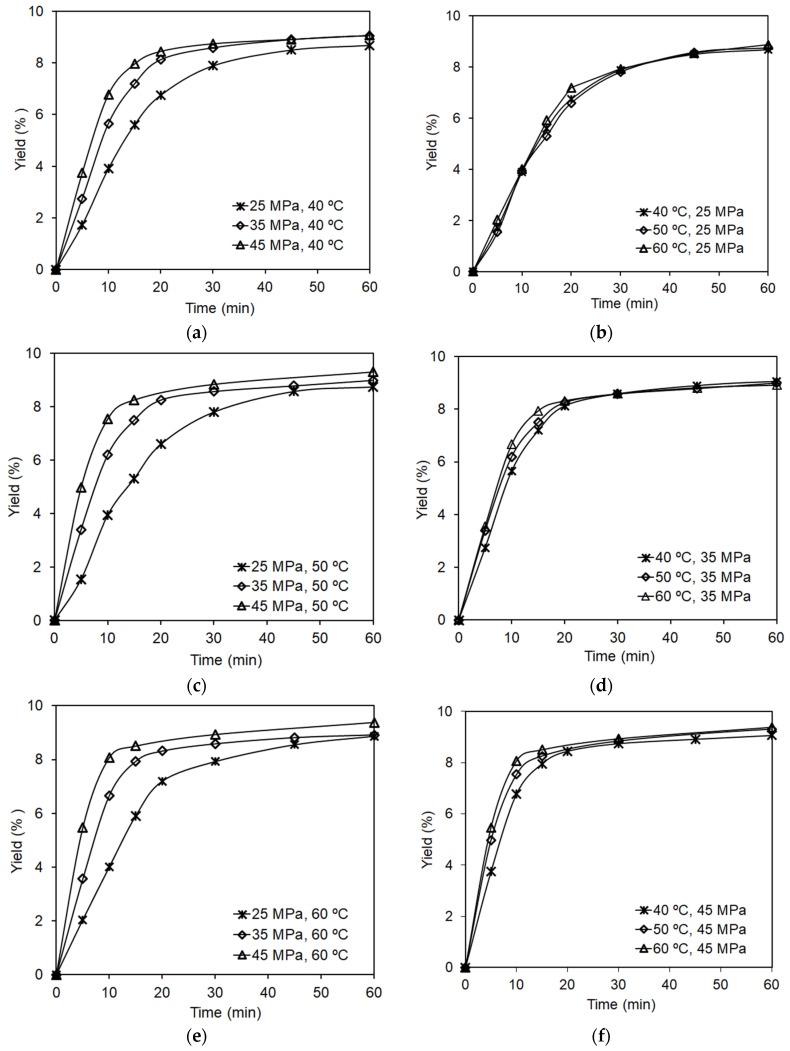
Kinetics of extraction of dry paprika using carbon dioxide depending on pressure and extraction temperature: (**a**) 40 °C; (**b**) 50 °C; (**c**) 60 °C; (**d**) 25 MPa; (**e**) 35 MPa; (**f**) 45 MPa.

The kinetic curves of the dry paprika extraction with SC-CO_2_ show that a significant part of the extract was recovered in the area of the linear dependence of yield on extraction time using a small amount of carbon dioxide (Figure 2). In the first 10 min of extraction, at the pressure of 45 MPa and the temperature of 50 °C (Figure 2b,f), approx. 80% of the total extract was obtained using only 17% of the total amount of CO_2_. However, at this stage, the carotenoid recovery was only 25%, and the carotenoid concentration in the extract was at a very low level. Depending on the extraction parameters applied, the total carotenoid content in extracts obtained during this stage was in the range of 0.5–1.5%. A similar phenomenon was observed by Ambrogi et al. [40]. In the first stage of the SFE process, they obtained approx. 75% extract using 15% of the total amount of solvent.

The analysis of kinetic curves of dry paprika extraction showed that pressure had the highest impact on the extraction rate and yield. The slope of the curves indicates a significant increase in the solubility of paprika extract in carbon dioxide with the increase in pressure at a constant temperature (Figure 2a–c). This could be due to the increase in solvent density and its dissolving power. Additionally, higher pressure can destroy the plant cell walls and stronger chemical interactions between different compounds and the structure of the plant cell walls, which can make it easier to extract components from the extraction bed. The effect of temperature on the solubility of the extract was much smaller than pressure (Figure 2d–f). At the same time, the increase in solubility with increasing pressure was higher at a higher temperature than that observed at a lower temperature. This is due to the solute vapor pressure that increases with temperature.

Based on the obtained kinetic curves, the apparent solubility of paprika extract in SC-CO_2_ was determined. The solubility of paprika extract at the temperature of 60 °C under the pressure of 25 MPa, 35 MPa, and 45 MPa was 4.8 g/kg, 9.4 g/kg, and 14.5 g/kg CO_2_, respectively. The calculated values of coefficients in Chrastil’s equation for the obtained data are as follows: k = 8.55, A = −5000, B = −40.67. These values were significantly higher than the values obtained during the determination of the solubility of paprika extract rich in carotenoids using the statistical method and were only slightly lower than those reported in the literature regarding the apparent solubility determined by the dynamic method [33]. Differences in solubility of paprika extract in SC-CO_2_ stem from the fact that the solubility depends on the chemical composition of the tested extract. Extracts obtained using a longer extraction time (in diffusion region) contain more compounds less soluble in carbon dioxide, which can result in decreased extract solubility.

### 3.2. Extraction Yield and Carotenoid Content in the Extract

The extract yield of Soxhlet extraction using n-hexane was 10.7%. The color value of the extract and the total carotenoid content in the extract obtained using n-hexane was 1510.0 ASTA and 4.42%, respectively.

The Soxhlet extraction yield in this work was higher than the yield obtained by Jaren-Galan et al. [24] (9.4%) and lower than the yield achieved by Fernández-Ronco et al. [21] after UAE with n-hexane (11.42%). However, the content of carotenoids and the color value of extract in the present study were the highest. This was due to the quality of the raw material and the extraction technique.

Table 1 presents the experimental plan matrix and the results obtained during the implementation of the research plan for the extraction of dry paprika with supercritical carbon dioxide. It should be noted that water was not found in the obtained extracts. It is a production waste and could affect the stability of the extract compounds. Additionally, all beds after the extraction of dry paprika were loose. The situation was different when paprika with a moisture content of 11% was extracted, which was described in a previous paper [34,38].

The yield of dry paprika extraction in the experimental area of independent variables was in the range of 5.10–10.23%. The highest yield was achieved after 60 min of extraction under the pressure of 45 MPa at the temperature of 60 °C using 50 kg CO_2_/kg feedstock (Run 4). The supercritical extraction efficiency was then 96% in reference to Soxhlet extraction using n-hexane.

The total content of carotenoids in the extract obtained from dry paprika ranged from 0.38% to 4.39%. The highest content of carotenoids was achieved for extract obtained after 110 min of extraction under the pressure of 45 MPa and temperature of 50 °C (Run 8). The color value of extract was then 1497 ASTA, and the carotenoid recovery was 90.9%. For the discussed experiment, the highest concentration of red carotenoids and a high concentration of yellow carotenoids was achieved. The carotenoid content in this extract was slightly lower than in the extract obtained using n-hexane Soxhlet extraction. Extract with the lowest concentration of carotenoids was obtained for extraction, which was carried out under the pressure of 25 MPa and temperature of 50 °C for 10 min (Run 3). The lowest recovery of carotenoids was then also noticed.

The analysis of kinetic curves of extraction (Figure 2) and data from Table 1 indicate that pressure and extraction time have the highest effect on the studied response variables. Statistical analysis of the second-order polynomial equations for the studied response variables was carried out to verify the above hypothesis, and regression coefficients were determined. The results of the statistical analysis for the full quadratic models are presented in Table 2.

The obtained regression models in the form of a second-order polynomial equation were statistically significant (*p* < 0.05) for the studied response variables (Y_e_, B_e_, C_k_, C_r_, C_y,_ and B_p_), and lack of fit was statistically insignificant (*p* > 0.05) (Table 2). The coefficient of determination R^2^ and adjusted coefficient of determination R^2^ were higher than 0.9. This means that the obtained models explain more than 90% of response value changes, indicating a high correlation between independent and response variables [41]. At the same time, the statistical analysis showed that some factors of the full quadratic model for the studied response variables are statistically insignificant (*p* > 0.05), and the obtained values of predicted R^2^ are relatively low. Therefore the reduction of models was carried out using the step-by-step method.

The reduction of statistically insignificant factors resulted in an improvement of the adjusted coefficient of determination R^2^ and a significant improvement of the predicted R^2^ for the studied models. A good agreement was also achieved between the predicted R^2^ and the adjusted coefficient of determination R^2^. The value of predicted R^2^ of reduced models for response variables Y_e_, B_e_, C_k_, C_r_, C_y_, and B_p_ increased to 0.86, 0.93, 0.93, 0.87, 0.96, and 0.89, respectively. The reduced regression models were highly statistically significant (*p* < 0.0001), and the lack of fit was statistically insignificant (*p* > 0.05). This means that the developed reduced models can be used to predict the values of response variables in the assumed range of independent factors variability.

Model significant terms for the yield of dry paprika extraction are linear terms of pressure (X_1_) and extraction time (X_3_), interactions of these two variables (X_1_X_3_), and quadratic terms of extraction time (X_3_^2^). The linear terms of temperature (X_2_) and quadratic terms of pressure (X_1_^2^) were also regarded as statistically significant, although they have a smaller effect on extraction yield, as reflected in the values of the regression coefficients. However, interactions between pressure and temperature (X_1_X_2_), interactions between temperature and time (X_2_X_3_), and quadratic terms of temperature (X_2_^2^) were removed from the model, as they had no significant effect on extraction yield. The RSM analysis confirmed the initial hypothesis that the pressure and time are highly statistically significant model parameters.

In the case of dry paprika extraction, the effect of input variables on the color value of extract, the total content of carotenoids in extract, and the concentration of red carotenoids in the extract was of a similar nature. These response variables were positively correlated with pressure (X_1_) and extraction time (X_3_). The quadratic terms of pressure (X_1_^2^) and quadratic terms of time (X_3_^2^) were also regarded as statistically significant. However, they were negatively correlated with discussed response variables. On the other hand, the linear terms of temperature (X_2_), quadratic terms of temperature (X_2_^2^) as well as interactions of the studied input variables turned out to be statistically insignificant (*p* > 0.05).

It can be concluded from the statistical analysis of regression coefficients that the concentration of yellow carotenoids in extract depends on the pressure (X_1_) and extraction time (X_3_). Moreover, quadratic terms of pressure (X_1_^2^) and time (X_3_^2^), as well as interactions of pressure and time (X_1_X_3_), were statistically significant. However, linear terms of temperature (X_2_), quadratic terms of temperature (X_2_^2^), interactions of pressure and temperature (X_1_X_2_), and interactions of temperature and time (X_2_X_3_) were regarded as statistically insignificant. The final models for response variables Y_e_, B_e_, C_k_, C_r_, C_y_, and B_p_ by eliminating the insignificant terms as a function of coded variables are presented below.

Y_e_ = 9.79 + 0.68 X_1_ + 0.25 X_2_ + 1.44 X_3_ − 0.72 X_1_X_3_ − 0.33 X_1_^2^ − 1.18 X_3_^2^ (2)

B_e_ = 1173.52 + 293.61 X_1_ + 507.52 X_3_ − 141.69 X_1_^2^ − 273.00 X_3_^2^ (3)

C_k_ = 3.44 + 0.86 X_1_ + 1.48 X_3_ − 0.41 X_1_^2^ − 0.80 X_3_^2^ (4)

C_r_ = 17.63 + 5.88 X_1_ + 8.74 X_3_ − 2.61 X_1_^2^ − 3.95 X_3_^2^ (5)

C_y_ = 12.92 + 1.88 X_1_ + 4.54 X_3_ − 0.59 X_1_X_3_ − 1.10 X_1_^2^ − 3.20 X_3_^2^ (6)

B_p_ = 28.06 − 26.32 X_1_ − 51.81 X_3_ + 25.25 X_1_^2^ + 33.82 X_3_^2^ (7)

**Figure 3:**
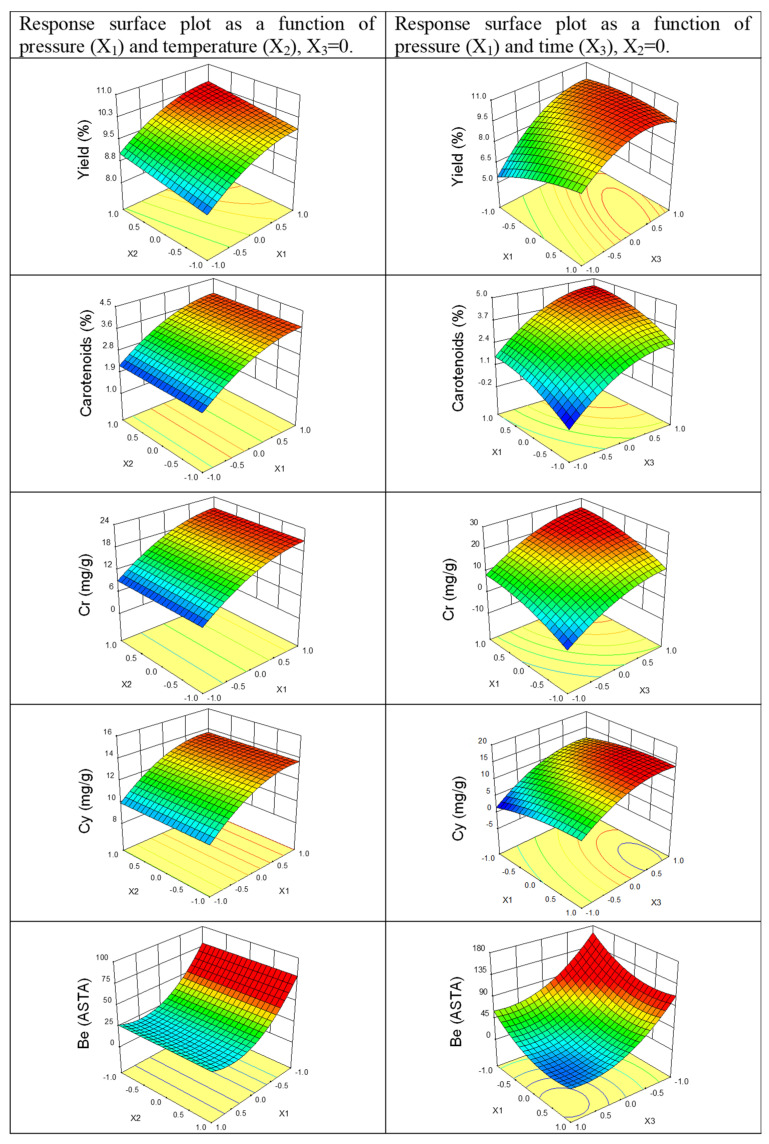
Diagrams of the response surface for response variables Y_e_, C_k_, C_r_, C_y_, and B_p_ in the function of extraction parameters. B_e_: color value of extract; C_r_: concentration of red carotenoid fraction in extract; C_y_: concentration of yellow carotenoid fraction in the extract.

The effects of process parameters on the studied response variables were presented as response surface diagrams (Figure 3). As seen in Figure 3, the yield of dry paprika extraction increases with increasing pressure, temperature, and extraction time. However, in this case, temperature has a much smaller effect on process yield than during the extraction of paprika with a moisture content of approx. 11% [38]. The experimental data show that the highest yield increase was observed when increasing the extraction time from 10 min to 70 min. A further increase in extraction time led to only an insignificant increase in yield. In the area of high yield increase, the consumption of carbon dioxide was from 8.3 to 58.3 kg CO_2_/kg feedstock.

The obtained results show that extraction pressure and time are important parameters influencing the total content of carotenoids in extract, the concentration of yellow and red carotenoids in the extract, and the color value of extract. The increased value of response variables C_k_, C_r_, C_y_, and B_e_ with the increase in pressure and time was, on the one hand, the result of an increase in the solubility of carotenoids in carbon dioxide related to the increasing solvent density and, on the other hand, a longer contact time between solute and solvent. As for extraction yield, an increase in extraction time from 10 to 70 min had a much higher effect on these response variables than after 70 min. Moreover, carotenoids isolated under lower pressure and with a shorter extraction time contained more fractions of yellow carotenoids, whereas, under higher pressure and longer time, they contained more fractions of red carotenoids [42]. After reducing the content of water in paprika by applying additional drying of the raw material, the temperature did not have a statistically significant effect on the content of carotenoids in the extract. This could be caused by removing the water barrier inhibiting diffusion of the solvent into the matrix and diffusion of the solutes outside the matrix.

As expected, the color value of post-extraction residue decreased with the increase in pressure (X_1_) and extraction time (X_3_). However, the quadratic terms of pressure (X_1_^2^) and time (X_3_^2^) were also regarded as statistically significant.

### 3.3. Process Optimization and Verification of Predicted Model

Based on the developed mathematical models, the optimal parameters were determined. In the first stage, optimization for each response variable was carried out individually by maximizing or minimizing its value. During optimization, independent variables X_1_ and X_3_ (pressure and extraction time) were assumed coded values from the entire studied variability range (from −1.0 to 1.0). For the output variables B_e_, C_k_, C_r_, and C_y_, the input variable X_2_ (extraction temperature) was minimized because, for all these response variables, the extraction temperature was not statistically significant. The optimal pressure, temperature, and time for maximizing the extraction yield were in the range of 29–45 MPa, 48–60 °C, 60–110 min, respectively, while the optimal conditions for maximizing response B_e_, C_k_, C_r_, and C_y_ were 38.7–45 MPa, 40 °C, 83–109.4 min; 38.8–44.9 MPa, 40 °C, 83–109.8 min; 41.6–45 MPa, 40 °C, 94.6–109.5 min; 35.2–44.8 MPa, 40 °C, 73.3–109.8 min, respectively. The optimal conditions for minimizing the color value of post-extraction residue were 32.8–45 MPa, 40.3–60 °C, and 71.8–110 min. As can be seen, the optimal conditions for the color value of extract were very similar to those for carotenoid content in extract.

At a further stage, multicriteria optimization using the desirability function was carried out. During optimization, the yield (Y_e_), the color value of extract (B_e_), and the total content of carotenoids in extract (C_k_) were maximized, and the value of post-extraction residue was minimized. The optimal parameters for the SC-CO_2_ extraction of dry paprika were: pressure of 45 MPa, temperature of 50 °C, and time of 74 min (61.5 kg CO_2_/kg feedstock). Under the optimal conditions, the predicted values of response Y_e_, B_e_, C_k_, C_r_, C_y_, and B_p_ were 10.24%, 1444.60 ASTA, 4.24%, 23.02 mg/g, 14.55 mg/g, and 15.27 ASTA, respectively. The desirability value was 0.97.

Three experiments of paprika extractions under optimal conditions were carried out to validate the developed mathematical models. Under the optimal conditions, the experimental values of response Y_e_, B_e_, C_k_, C_r_, C_y_, and B_p_ were 10.05%, 1437.00 ASTA, 4.21%, 23.85 mg/g, 13.73 mg/g, and 18.60 ASTA, respectively. As it can be observed, the obtained experimental data are compliant with the predicted values and fell within limits set by relevant confidence intervals. This confirms the accuracy of the assumed mathematical models and reliability of BBD coupling and the desirability function method.

### 3.4. Content of Fatty Acids

The fatty acid content was studied to assess the quality attributes of paprika extracts. Table 3 contains the fatty acid profile of extracts obtained by SC-CO_2_ under optimal conditions (Figure S1) and the Soxhlet technique with n-hexane (Figure S2). The fatty acid profile of paprika extract revealed the presence of palmitic, stearic, behenic, lauric, arachidic, linoleic, α-linoleic, and oleic acid.

The content of saturated fatty acids (SFAs) in the extract obtained using SC-CO_2_ was 13.39%, and it was slightly lower than in the extract obtained using the Soxhlet technique. Paprika extract is made up of a higher proportion of unsaturated fatty acids (UFAs) than SFAs. The UFAs in the extract obtained using SC-CO_2_ accounted for ≈81% of the total amount of fatty acids identified in extracts (≈12% monounsaturated fatty acids and ≈69% polyunsaturated fatty acids).

Table 3 shows that the major fatty acids contained in paprika extract are linoleic, palmitic, and oleic acid, which is compliant with the literature reports presented by El-Adawy and Taha and Asilbekova [43,44]. The identified oleic acid is particularly important because of its health-promoting properties [45]. It can help reduce the risk of heart diseases by raising levels of high-density lipoprotein and lowering low-density lipoprotein. The high content of linoleic and α-linoleic acids also contributes to the enhancement of the nutritional value of the obtained extract.

### 3.5. Stability of Carotenoids in Paprika Extract

Paprika extract is widely used as a source of natural colorants, including bioactive compounds. The extract stability affects its use in industries. Therefore, it is of special interest from the technological and commercial points of view to minimize the loss of carotenoids during processing and storage.

**Figure 4:**
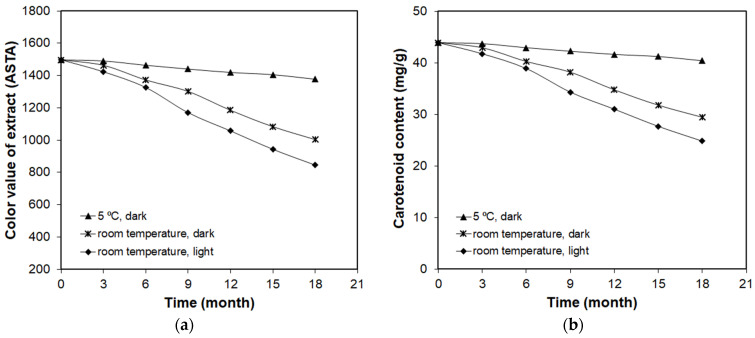
Effect of conditions and storage time on: (**a**) color value of extract; (**b**) total content of carotenoids in paprika extract.

To determine the effect of storage conditions and time on the stability of carotenoids, the paprika extract obtained with SC-CO_2_ was divided into three samples and placed in dark and air-tight glass bottles. One set was kept in daylight at room temperature, and the second bottle was kept at room temperature protected from daylight (dark condition), whereas the third one was additionally covered with aluminum foil and kept at temperature 5 °C. At constant time intervals (three months), the color value of extract and total content of carotenoids in extracts were determined. Changes in the color value of extract and the total content of carotenoids in extract depending on time and conditions of storage are presented in Figure 4.

The research showed that storage conditions and time have an effect on the color value and the content of carotenoids in the extract, and the color changes of paprika extract correlated well with carotenoid content. Samples that were exposed to light showed a greater reduction in the carotenoid content and color value of extract as compared to samples kept in the dark. This is related to the fact that carotenoids can undergo photodegradation and isomerization under the effect of light [46]. The carotenoids in the extracts can also undergo indirect oxidation through fatty acids oxidation.

The results clearly showed the immense effect of storage temperature on the stability of the carotenoid compounds. The content of carotenoids decreased from 43.90 to 40.39 and 29.42 mg/g after storage for 18 months at 5 °C and room temperature, respectively. The most significant changes were observed for extract kept at ambient temperature and exposed to daylight. Then, during a year of storage, almost 30% losses of carotenoids were observed. Limiting access to light reduced the carotenoid losses to 20%. The smallest changes were observed for paprika extract stored at 5 °C without access to daylight. After one year of storage, the loss of carotenoids amounted only to 5%.

## 4. Conclusions

The Box–Behnken design (BBD) and response surface method (RSM) were applied to study the effect of major process parameters and their interactions on the SC-CO_2_ extraction of dry paprika and determine the optimal conditions. The statistical analysis showed that pressure, temperature, and extraction time had a significant effect on the extraction yield. Moreover, the quadratic term of pressure and the quadratic term of extraction time, as well as the interactions between pressure and time, were statistically significant. At the same time, no statistically significant effect of the extraction temperature on the quality of paprika extract was found (the color value of extract, the total content of carotenoids in extract, the concentration of yellow and red carotenoids in extract). Highly statistically significant mathematical models were obtained for the studied response, i.e., variables with a high coefficient of determination R^2^, adjusted R^2^, and predicted R^2^. The optimal extraction parameters were specified with the use of validated models: pressure of 45 MPa, temperature of 50 °C, and time of 74 min. In these conditions, the extract recovery was 94%, and the recovery of carotenoids was 87%. The obtained extract was rich in unsaturated fatty acids amounting to 56%. Storage of paprika extract at 5 °C without light access minimizes carotenoid losses and extends its storage time and use.

## Figures and Tables

**Table 1:** Actual-coded independent variables with assumed levels of the BBD design and results of SC-CO_2_ extraction of dry paprika.

Run Order	Independent Variables						Dependent Variables					
	Pressure		Temperature		Time		Y_e_ (%)	B_e_ (ASTA)	C_k_ (%)	C_r_ (mg/g)	C_y_ (mg/g)	B_p_ (ASTA)
	X_1_	p (MPa)	X_2_	T (°C)	X_3_	t (min)						
1	1	45	0	50	−1	10	8.43	508.4	1.49	6.33	6.84	124.12
2	−1	25	−1	40	0	60	8.93	743.2	2.18	8.97	10.25	85.00
3	−1	25	0	50	−1	10	5.10	130.4	0.38	1.07	2.21	165.14
4	1	45	1	60	0	60	10.23	1400.2	4.11	22.53	14.02	15.42
5	0	35	1	60	1	110	10.12	1442.1	4.20	22.80	14.55	12.15
6	0	35	−1	40	1	110	9.88	1478.7	4.34	24.27	14.46	16.63
7	0	35	0	50	0	60	9.63	1149.0	3.37	16.74	13.06	22.00
8	1	45	0	50	1	110	10.00	1496.8	4.39	25.08	14.06	13.00
9	0	35	0	50	0	60	9.84	1222.8	3.59	18.70	13.27	36.93
10	−1	25	0	50	1	110	9.57	931.2	2.73	12.30	11.78	49.00
11	−1	25	1	60	0	60	9.17	602.1	1.77	6.32	9.15	87.00
12	0	35	0	50	0	60	9.85	1180.3	3.46	17.95	12.85	28.00
13	0	35	1	60	−1	10	7.69	324.8	0.95	3.41	4.85	129.34
14	0	35	−1	40	−1	10	6.81	325.0	0.95	3.74	4.62	86.63
15	1	45	−1	40	0	60	9.57	1350.3	3.96	21.77	13.48	23.04

Y_e_: extraction yield; B_e_: color value of extract; C_k_: total content of carotenoids in extract; C_r_: concentration of red carotenoid fraction in extract; C_y_: concentration of yellow carotenoid fraction in extract; B_p_: color value of post-extraction residue.

**Table 2:** Regression coefficients for full quadratic model and model parameters.

Source	Y_e_ (%)		B_e_ (ASTA)		C_k_ (%)		C_r_ (mg/g)		C_y_ (mg/g)		B_p_ (ASTA)	
	β	*p*-Value	β	*p*-Value	β	*p*-Value	β	*p*-Value	β	*p*-Value	β	*p*-Value
Model		0.0011		0.0008		0.0008		0.0025		<0.0001		0.0022
Lack of Fit		0.0757		0.0692		0.0731		0.0983		0.0995		0.1723
β_0_	9.79		1184.03		3.47		17.80		13.06		28.98	
X_1_	0.68	0.0026	293.60	0.0006	0.86	0.0006	5.88	0.0010	1.88	0.0001	−26.32	0.0029
X_2_	0.25	0.0950	−16.00	0.6937	−0.05	0.6721	−0.46	0.6134	−0.03	0.8747	4.08	0.4418
X_3_	1.44	<0.0001	507.53	<0.0001	1.49	<0.0001	8.74	0.0002	4.54	<0.0001	−51.81	0.0001
X_1_X_2_	0.11	0.5720	47.75	0.4187	0.14	0.4145	0.85	0.5130	0.41	0.1697	−2.41	0.7418
X_1_X_3_	−0.73	0.0087	46.90	0.4265	0.14	0.4224	1.88	0.1814	−0.59	0.0699	1.26	0.8629
X_2_X_3_	−0.16	0.3994	−9.10	0.8733	−0.04	0.8328	−0.29	0.8233	−0.04	0.8965	−11.80	0.1482
X_1_^2^	−0.33	0.1330	−143.02	0.0522	−0.42	0.0522	−2.63	0.0914	−1.12	0.0085	25.13	0.0173
X_2_^2^	0.03	0.8920	−17.07	0.7745	−0.05	0.7598	−0.27	0.8391	−0.22	0.4485	−1.49	0.8434
X_3_^2^	−1.17	<0.0013	−274.32	0.0046	−0.81	0.0043	−3.97	0.0254	−3.22	<0.0001	33.71	0.0054
R^2^		0.9792		0.9814		0.9818		0.9711		0.9945		0.9727
Adjusted R^2^		0.9419		0.9480		0.9489		0.9190		0.9846		0.9235
Predicted R^2^		0.6825		0.7147		0.7206		0.5636		0.9171		0.6073

β is coefficient of equation; Y_e_: extraction yield; B_e_: color value of extract; C_k_: total content of carotenoids in extract; C_r_: concentration of red carotenoid fraction in extract; C_y_: concentration of yellow carotenoid fraction in extract; B_p_: color value of post-extraction residue; *p* < 0.0001, very highly significant, *p* < 0.01, very significant, *p* < 0.05, significant, *p* > 0.10, not significant.

**Table 3:** Composition and content of fatty acids of paprika extract.

Fatty Acids (%)	Method of Extraction	
	SC-CO_2_ (45 MPa, 50 °C) ^a^	SOX ^a^
Lauric acid (C12:0)	0.43 ± 0.11	0.83 ± 0.18
Palmitic acid (C16:0)	10.68 ± 0.23	10.46 ± 0.21
Stearic acid (C18:0)	1.86 ± 0.01	1.84 ± 0.03
Oleic acid (C18:1)	8.23 ± 0.10	7.86 ± 0.20
Linoleic acid (C18:2)	44.87 ± 0.33	42.91 ± 0.32
Arachidic acid (C20:0)	0.26 ± 0.01	0.28 ± 0.01
α-Linolenic acid (C18:3)	2.86 ± 0.18	3.54 ± 0.15
Behenic acid (C22:0)	0.16 ± 0.01	0.18 ± 0.01
Total saturated fatty acids	13.39	13.59
Total unsaturated fatty acids	55.96	54.31

^a^ Each value is the mean ± standard deviation of triplicate determinations; SC-CO_2_: extract obtained by supercritical carbon dioxide extraction; SOX: extract obtained by Soxhlet extraction.

## Data Availability

All data analyzed during this study are included in this article.

## Supplementary Materials

The following supporting information can be downloaded at: https://www.mdpi.com/article/10.3390/molecules27072090/s1, Figure S1: Chromatogram of paprika extract obtained by supercritical carbon dioxide; Figure S2: Chromatogram of paprika extract obtained by Soxhlet extraction method.
