# Supplementary material for: Pilot-Scale Optimization of Supercritical CO2 Extraction of Dry Paprika Capsicum annuum: Influence of Operational Conditions and Storage on Extract Composition
Source: Molecules. 2022 Mar 24;27(7):2090. doi: 10.3390/molecules27072090 (PMC9000775; doi:10.3390/molecules27072090)
Supplement: Supplementary file 1 [file molecules-27-02090-s001.zip › molecules-1603054-supplementary.pdf]

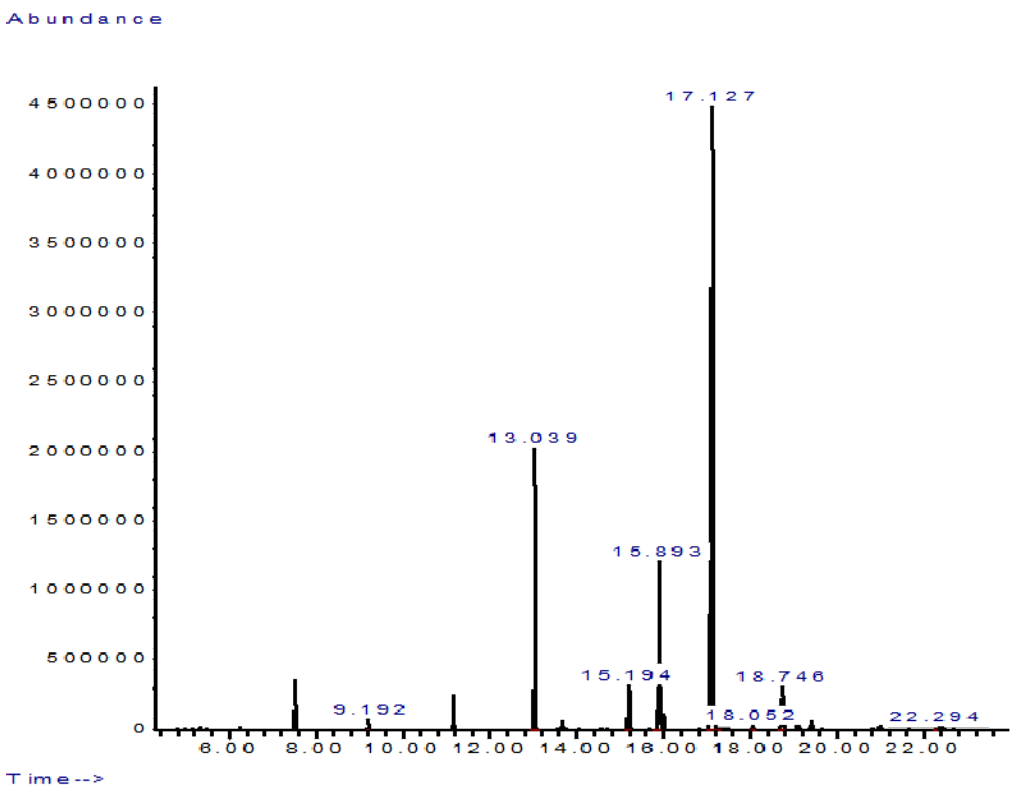

Figure S1. Chromatogram of paprika extract obtained by supercritical carbon dioxide (GCMSD technique; Retention time RT [min]: 9.19 C12:0; 13.04 C16:0; 15.19 C18:0; 15.89 C18:1; 17.13 C18:2; 18.05 C20:0; 18.75 C18:3; 22.29 C22:0).

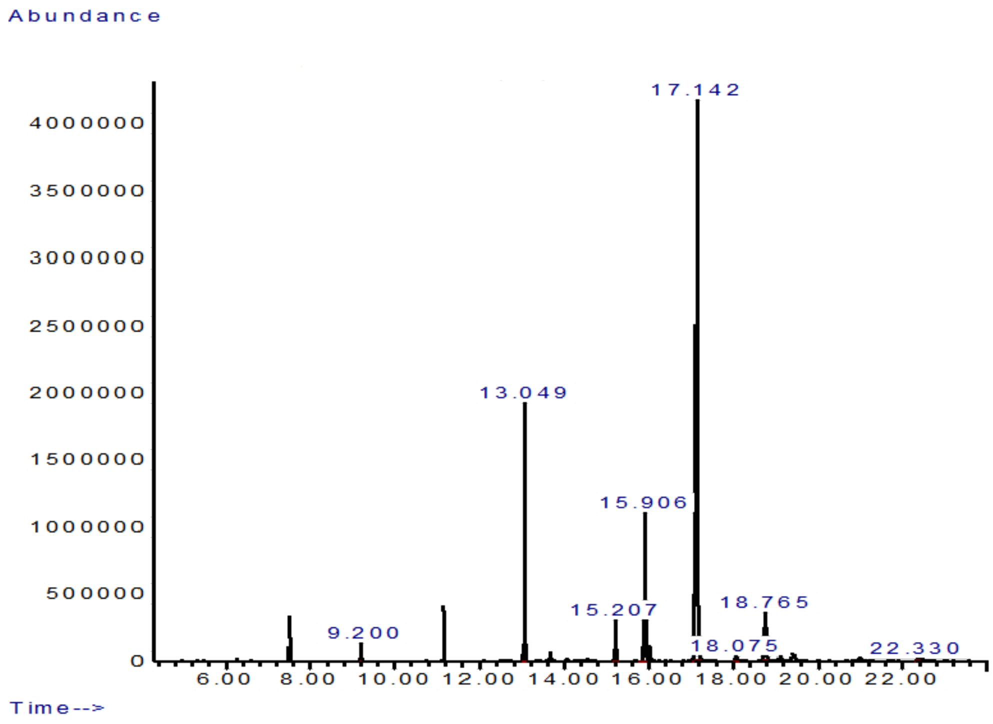

Figure S2. Chromatogram of paprika extract obtained by Soxhlet extraction method (GCMSD technique; Retention time RT [min]: 9.20 C12;0; 13.05 C16:0; 15.21 C18:0; 15.91 C18:1; 17.14 C18:2; 18.08 C20:0; 18.77 C18:3; 22.33 C22:0).
